# Characterization of 2-phenanthroate:CoA ligase from the sulfate-reducing, phenanthrene-degrading enrichment culture TRIP

**DOI:** 10.1128/aem.01296-24

**Published:** 2024-09-09

**Authors:** I. Kaplieva-Dudek, Nadia A. Samak, Jenny Bormann, Farnusch Kaschani, Markus Kaiser, Rainer U. Meckenstock

**Affiliations:** 1 Environmental Microbiology and Biotechnology (EMB), Aquatic Microbiology, Faculty of Chemistry, University of Duisburg-Essen, Essen, Germany; 2 Department of Chemical Biology, ZMB, Faculty of Biology, University of Duisburg-Essen, Essen, Germany; 3 Analytics Core Facility Essen, ZMB, Faculty of Biology, University of Duisburg-Essen, Essen, Germany; Shanghai Jiao Tong University, Shanghai, China

**Keywords:** aryl-CoA ligase, 2-phenanthroic acid, anaerobic phenanthrene degradation, PAH, oil degradation

## Abstract

Polycyclic aromatic hydrocarbons (PAHs) are chemically stable pollutants that are poorly degraded by microorganisms in anoxic sediments. The anaerobic degradation pathway of PAHs such as phenanthrene starts with a carboxylation reaction forming phenanthroic acid. In this study, we identified and characterized the next enzyme in the pathway, the 2-phenanthroate:CoA ligase involved in the ATP-dependent formation of 2-phenanthroyl-CoA from cell-free extracts of the sulfate-reducing enrichment culture TRIP grown anaerobically with phenanthrene. The identified gene sequence indicated that 2-phenanthroate:CoA ligase belongs to the phenylacetate:CoA ligase-like enzyme family. Based on the sequence, we predict a two-domain structure of the 2-phenanthroate:CoA ligase with a typical large N-terminal and a smaller C-terminal domain. Partial purification of 2-phenanthroate:CoA ligase allowed us to identify the coding gene in the genome. 2-Phenanthroate:CoA ligase gene was heterologously expressed in *Escherichia coli*. Characterization of the 2-phenanthroate:CoA ligase was performed using the partially purified enzyme from cell-free extract and the purified recombinant enzyme. Testing all possible phenanthroic acid isomers as substrate for the ligase reaction showed that 2-phenanthroic acid is the preferred substrate and only 3-phenanthroic acid can be utilized to a minor extent. This also suggests that the product of the prior carboxylase reaction is 2-phenanthroic acid. 2-Phenanthroate:CoA ligase has an optimal activity at pH 7.5 and is oxygen-insensitive, analogous to other aryl-CoA ligases. In contrast to aryl-Coenzyme A ligases reported in the literature, which need Mg^2+^ as cofactor, 2-phenanthroate:CoA ligase showed greatest activity with a combination of 5 mM MgCl_2_ and 5 mM KCl. Furthermore, a substrate inhibition was observed at ATP concentrations above 1 mM and the enzyme was also active with ADP.

**IMPORTANCE:**

Polycyclic aromatic hydrocarbons (PAHs) constitute a class of very toxic and persistent pollutants in the environment. However, the anaerobic degradation of three-ring PAHs such as phenanthrene is barely investigated. The initial degradation step starts with a carboxylation followed by a CoA‑thioesterification reaction performed by an aryl-CoA ligase. The formation of a CoA-thioester is an important step in the degradation pathway of aromatic compounds because the CoA-ester is needed for all downstream biochemical reactions in the pathway. Furthermore, we provide biochemical proof for the identification of the first genes for anaerobic phenanthrene degradation. Results presented here provide information about the biochemical and structural properties of the purified 2‑phenanthroate:CoA ligase and expand our knowledge of aryl-CoA ligases.

## INTRODUCTION

**Fig 1:**
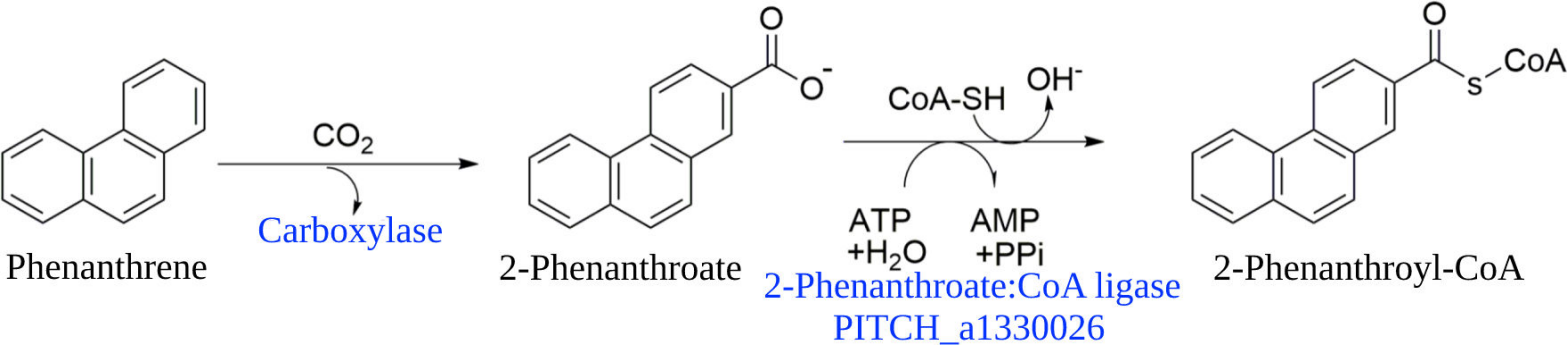
Initial steps of anaerobic phenanthrene degradation to 2-phenanthroyl-CoA by sulfate-reducing microorganisms. Phenanthrene is activated to 2-phenanthroic acid by carboxylation and further converted to 2-phenanthroyl-CoA by an ATP-dependent 2-phenanthroate:CoA ligase.

Polycyclic aromatic hydrocarbons (PAHs) are hazardous pollutants that occur in the environment due to anthropogenic activities such as petroleum spills and incomplete combustion processes (1–3) or natural processes such as wood fires (4, 5). PAHs are chemically stable, poorly soluble, and consequently accumulate in soils, sediments, or groundwater and especially in anoxic zones (1, 3, 6, 7). Only microorganisms are able to completely biodegrade PAHs to CO_2_ which is well-studied for aerobic degradation pathways (8–10). However, degradation of PAHs under anoxic conditions is an extremely slow process. So far, the anaerobic degradation pathways of PAHs have been only partially elucidated for naphthalene (11–13), 2-methylnaphthalene (12, 14, 15), and even less for phenanthrene (16–20). The initial step of naphthalene and phenanthrene degradation by sulfate-reducing microorganisms is a carboxylation reaction followed by the formation of a CoA-thioester (Fig. 1) (12, 18). Thioester formation might facilitate the uptake of aromatic hydrocarbons (21–23), but it is also crucial for further degradation (24–27).

In bacteria metabolizing monocyclic aromatic compounds, this CoA-thioesterification reaction is predominantly catalyzed by aryl-CoA ligases belonging to class I of the adenylate-forming enzyme (ANL) superfamily. Further members of the class I ANL superfamily are acyl-CoA synthetases/ligases, the adenylation domain of non-ribosomal peptide synthetases and luciferases. They all show low sequence similarity (between 20% and 30%), but high structural homology (28, 29). CoA-ligases which are expected to be involved in the anaerobic degradation of PAHs such as phenanthrene have not yet been characterized, but a putative CoA-ligase activity was detected in cell-free extracts of the phenanthrene-degrading enrichment culture TRIP (18).

The sulfate-reducing, phenanthrene-degrading culture TRIP was enriched from soil of the largest natural asphalt lake, Pitch Lake in Trinidad (18, 30). The culture grows very slowly and reaches only faint turbidity of 10^6^ cells mL^−1^ after 6–12 months of cultivation. Nevertheless, in the attempt to elucidate the anaerobic degradation pathway of phenanthrene in a proteogenomic study, we were able to identify proteins and genes involved in the degradation (20). The putative genes for anaerobic phenanthrene degradation were homologous to genes known from anaerobic naphthalene degradation by the sulfate-reducing culture N47 (31) and NaphS2 (32) and were located on the metagenome of the candidate species *Desulfatiglans* TRIP_1, a sulfate-reducer which constituted up to 90% of all cells of the enrichment (20).

In this work, 2-phenanthroate:CoA ligase was partially purified by ammonium-sulfate precipitation and hydrophobic interaction chromatography (HIC) and analyzed via bottom-up proteomics by LC/MS/MS. The identified amino acid sequence of 2-phenanthroate:CoA ligase was compared with previously known sequences of aryl-CoA ligases to predict the structure and function of 2-phenanthroate:CoA ligase. The characterization was performed from cell-free extracts (cfe) of the anaerobic, phenanthrene-degrading, sulfate-reducing enrichment culture TRIP but also with purified enzyme that was heterologously expressed in *Escherichia coli* (*E. coli*).

## RESULTS

### Enrichment and identification of 2-phenanthroate:CoA ligase

**Fig 2:**
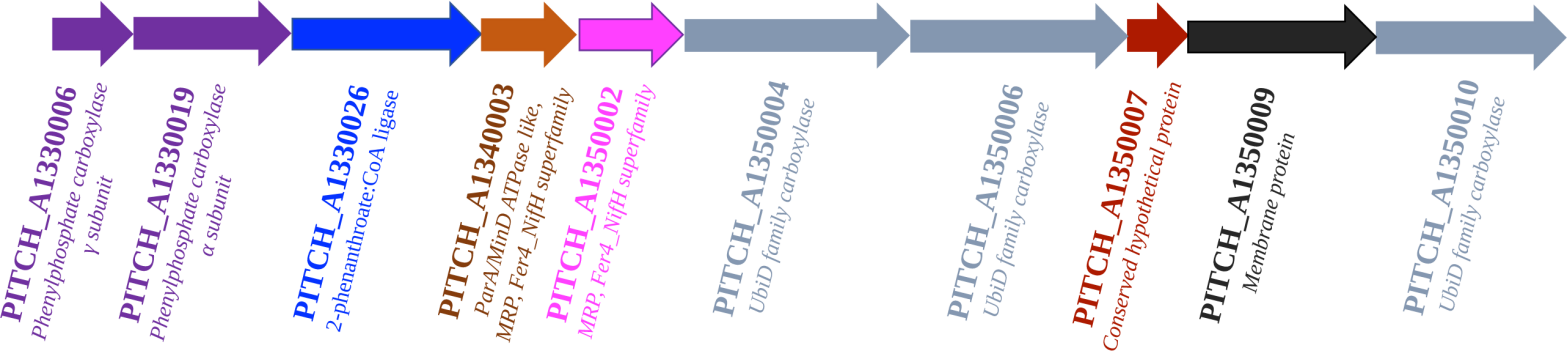
Gene cluster potentially encoding enzymes for phenanthrene carboxylation and for the identified 2-phenanthroate:CoA ligase.

2-Phenanthroate:CoA ligase was enriched from 1.5 L of culture TRIP grown anaerobically with phenanthrene and subsequently identified by proteomic analysis. Since 2-phenanthroate:CoA ligase was oxygen insensitive and showed no significant loss of activity after freezing at −20°C, all protein fractions could be stored at −20°C until the next purification step or enzymatic assay. The purification procedure started with 2.5 M ammonium sulfate precipitation of 1.0 mL cell extract containing 2.99 ± 1.37 mg of proteins. The resulting pellet was then dissolved in 2 M ammonium sulfate, loaded on a HIC column and eluted in a gradient from 2 M to 0 M ammonium sulfate. The procedure resulted in a purification factor of 11.1 ± 1.0 with a yield of 50% and a specific activity of 116.7 ± 17.1 mU mg^−1^ protein (Table 1). Due to the very low biomass yield of the culture, only 39.8 ± 29.6 µg of enriched 2-phenanthroate:CoA ligase was recovered from the low initial amount of protein (2.99 ± 1.37 mg from 1.5 L of culture), which made further purification steps impossible. After purification with ammonium sulfate precipitation and HIC, SDS-PAGE indicated a barely visible band around 53 kDa indicating the potential 2-phenanthroate:CoA ligase enzyme and other bands indicating impurities (Fig. S1). The enrichment of the 2-phenanthroate:CoA ligases performed in this study includes the one encoded by the gene PITCH_a1330026. In an earlier proteogenomic study, it was found that three putative aryl-CoA ligases were expressed by the candidate *Desulfatiglans* TRIP_1 during growth with phenanthrene and encoded by the genes PITCH_a1330026, PITCH_a1100006, and PITCH_a760031 (20). These genes are located in the vicinity of the putative carboxylase gene cluster (20) supporting their role in phenanthrene metabolism (Fig. 2).

**TABLE 1:** Purification protocol of 2-phenanthroate:CoA ligase from TRIP culture grown with phenanthrene

Step	Total protein (µg)	Activity (mU)	Specific activity (mU/mg)	Yield (%)	Purification (*x*-fold)
Cell-free extract	2,290 ± 1,372	0.4 ± 0.1	11.6 ± 2.8	100	1
Ammonium sulfate	1,279 ± 659	0.4 ± 0.1	27.2 ± 8.5	100	2.9 ± 1.4
HIC	39.8 ± 29.6	0.2 ± 0.1	116 ± 17.1	50	11.1 ± 1.0

### Sequence comparison and characterization

Homologous proteins of 2-phenanthroate:CoA ligase (encoded by PITCH_a1330026) were searched in the NCBI and PDB databases via the program BLAST (REF). The strongest hits were obtained with the phenylacetate:CoA ligases, PaaK1 and PaaK2, from *Burkholderia cenocepacia* J2315 (33), with sequence identities of 31.28% and 29.55%, respectively and sequence coverage of 95%. The next highest sequence similarity (28.94%, sequence coverage of 95%) was found with the 2-hydroxyisobutyric acid:CoA ligase from the MTBE-degrading bacterium *Aquincola tertiaricarbonis* strain L108 (34). Surprisingly, the ligase AjiA1 from *Streptomyces* sp. catalyzing the ATP-dependent dimerization of 3-hydroxyanthranilic acid (35) was also identified by the PDB database with 90% sequence coverage and sequence similarity of 23.71% with 2-phenanthroate:CoA ligase.

Multiple sequence alignment of these sequences and 14 representatives of the aryl-CoA ligase family revealed four of the conserved motifs common to the adenylate-forming enzyme superfamily (36) including the P-loop (A3/3), adenine-binding motif (A5), and linker motif (A8) (Fig. S2). Furthermore, 6 amino acids were invariant among all the aligned sequences, G_210_, G_213_, P_215_, P_304_, R_465_, and K_566_ (residue positions correspond to the multiple sequence alignment of this paper), and 12 amino acids were found identical with the 2-phenanthroate:CoA ligase (encoded by PITCH_a1330026) sequence in at least 70% of all sequences. Thirteen amino acids were only identified invariant in the phenylacetate:CoA ligase-like group.

**Fig 3:**
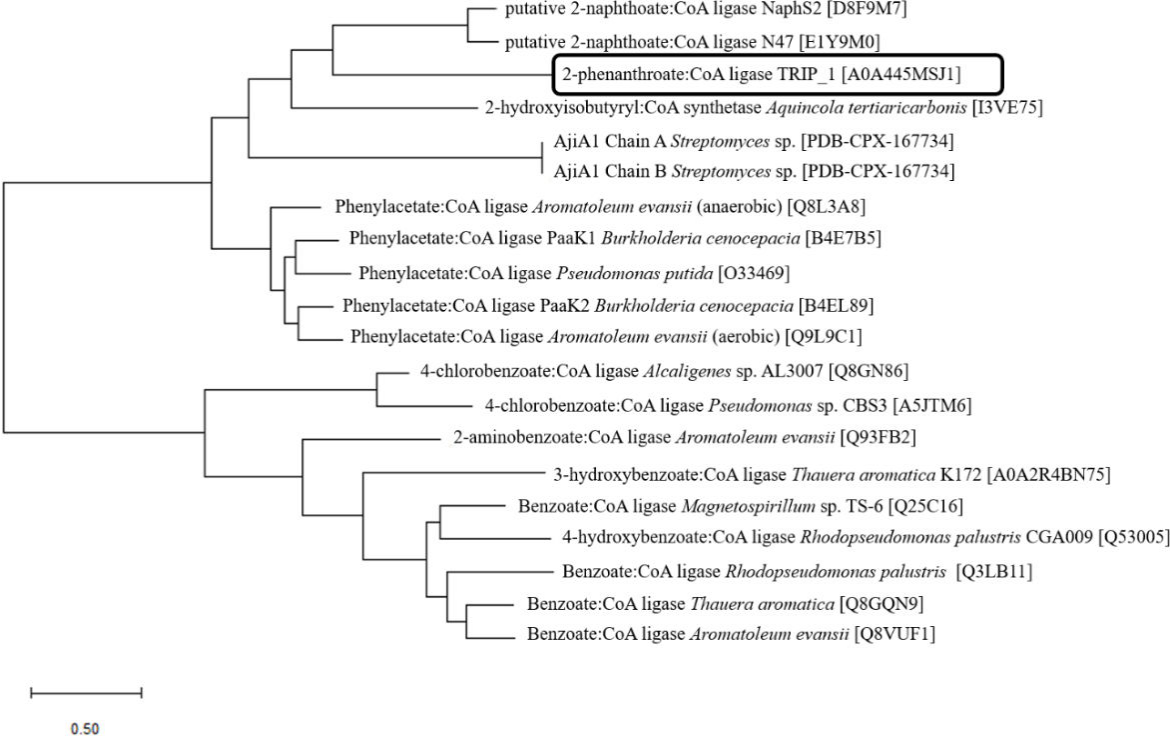
Maximum-likelihood phylogenetic tree of 2-phenanthroate:CoA ligase, 16 representative aryl-CoA ligases, 2-hydroxyisobutyryl-CoA synthetase and AjiA1. The tree was constructed in Mega-X using the corresponding amino acid sequences (37, 38).

The phylogenetic analysis using maximum-likelihood method underlines that 2-phenanthroate:CoA ligase (encoded by PITCH_a1330026) belongs to the phenylacetate:CoA ligase-like enzyme family (Fig. 3).

### Heterologous expression of the gene encoding 2-phenanthroate:CoA ligase

**Fig 4:**
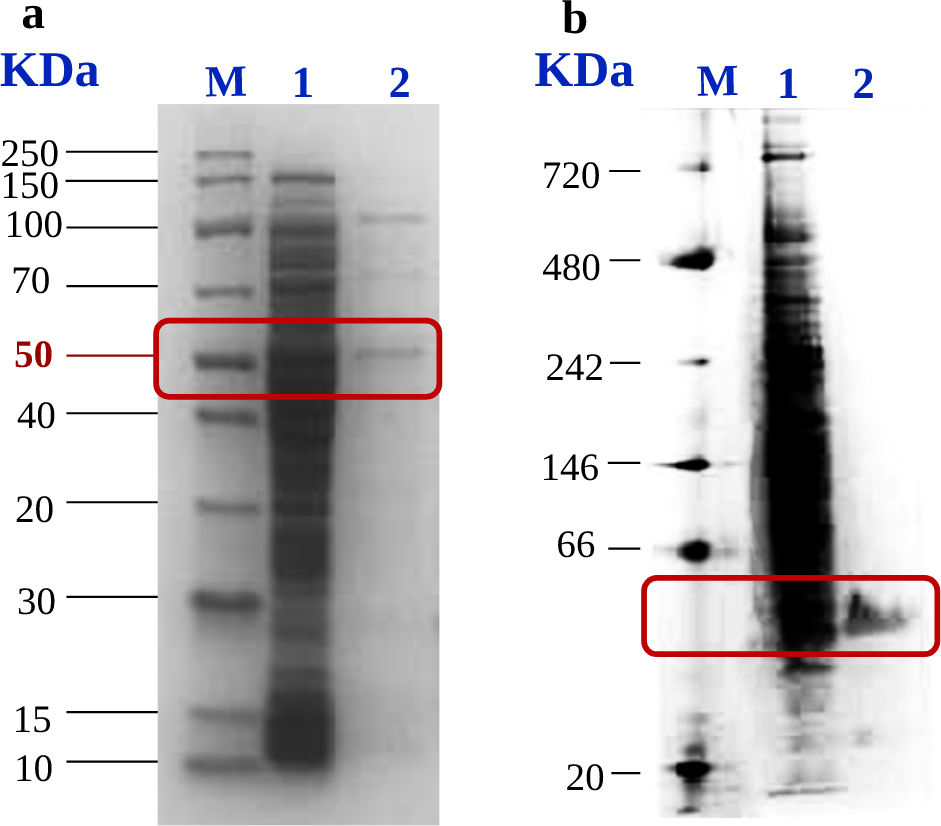
SDS-PAGE analysis (a) and NATIVE-PAGE analysis (b) of the heterologously expressed 2-phenanthroate:CoA ligase. M, protein MW standard; lane 1, *E. coli* cell-free extract; lane 2, ligase after purification with Strep-Tactin resin.

Putative 2-phenanthroate:CoA ligases encoded by the genes PITCH_a1330026, PITCH_a1100006, and PITCH_a760031 were heterologously expressed to overcome the very low yield of the purified enzyme from TRIP cell-free extract. Soluble 2-phenanthroate:CoA ligase was expressed from *E. coli* carrying the recombinant plasmid PASG-IBA103-PITCH_a1330026 with C-terminal twin-Strep-tag. 0.5 mg protein was produced from 8 g *E. coli* cells wet weight. The heterologous expression of 2-phenanthroate:CoA ligase from *E. coli* was confirmed by SDS-PAGE, revealing the expression of the ≈53 kDa protein (Fig. 4a). Other protein bands present in the gel were impurities. The size and purity of the expressed enzyme were also confirmed by the Native-PAGE analysis which showed a monomeric protein with a size of ≈53 kDa (Fig. 4b). The other two aryl-CoA ligases were also heterologously expressed using the same expression vector, but the two enzymes were inactive towards phenanthroic acid (data not shown). Therefore, the active 2-phenanthroate:CoA ligase encoded by the gene PITCH_a1330026 is the only enzyme characterized in this study.

### Substrate specificity of phenanthroate:CoA ligase

Phenanthroate:CoA ligase was characterized using TRIP cell-free extract, primarily due to the markedly low yield of the enzyme purified from cell-free extracts. Additionally, characterization of the enzyme was also performed using the purified recombinant enzyme. The substrate specificity of phenanthroate:CoA ligase toward phenanthroic acid as a key intermediate in the phenanthrene degradation by sulfate-reducing microorganisms was tested in an anaerobic ligase assay with cell-free extract using all possible isomers of phenanthroic acid (Sigma-Aldrich, USA). Production of the corresponding phenanthroyl-CoA from phenanthroic acid and free CoA was detected via LC-MS (Table 2). 2-Phenanthroic acid was converted best with a specific activity of 2,500 nmol min^−1^ mg^−1^. With 3-phenanthroic acid as substrate, the enzyme activity was only 20% compared to 2-phenanthroic acid. A much lower production of 1-phenanthroyl-CoA and 4-phenanthroyl-CoA was detected, and no corresponding CoA-thioester product was observed for the substrate 9-phenanthroic acid. These results indicate that the ligase present in TRIP culture appears to prefer 2-phenanthroic acid as substrate, and we term this enzyme 2-phenanthroate:CoA ligase, accordingly. The purified 2-phenanthroate:CoA ligase from *E. coli* showed the same characteristics as the enzyme purified from the TRIP culture and under oxic conditions. The enzyme converted 2-phenanthroic acid and 3-phenanthroic acid to 2-phenanthroyl-CoA and 3-phenanthroyl-CoA with a specific activity of 180 and 2.4 nmol min^−1^ mg^−1^, respectively. Activity toward other substrates (4-phenanthroic acid, 1-phenanthroic acid, and 9-phenanthroic acid) was not detected, confirming that 2-phenanthroate:CoA ligase prefers 2-phenanthroic acid as substrate. The difference between the cell-free extract and the pure recombinant enzyme in the utilization of the substrates 4-phenanthroic acid and 1-phenanthroic acid could be attributed to the presence of other aryl-CoA ligases in the cell-free extract. However, the conversion is very low when compared with the conversion of 2-phenanthroic acid.

**TABLE 2:** Catalytic properties of 2-phenanthroate:CoA ligase from culture TRIP and the purified recombinant enzyme from *E. coli^a^*

Parameter	Specific activity of phenanthroate:CoA ligase (nmol min^−1^ mg^−1^)	
	Cell-free extract	Heterologously expressed
Substrate		
1 mM 2-phenanthroic acid	2,482 ± 526	180.4 ± 30.8
1 mM 3-phenanthroic acid	518 ± 89	2.4 ± 0.19
1 mM 4-phenanthroic acid	27 ± 1	ND
1 mM 1-phenanthroic acid	22 ± 5	ND
1 mM 9-phenanthroic acid	0	ND
Nucleotide		
5 mM ADP	5,783 ± 1,279	152.1 ± 26.7
5 mM ATP	4,914 ± 303	203.8 ± 15.9
5 mM CTP	2,578 ± 1,262	2.1 ± 0.1
5 mM UTP	1,192 ± 880	0.67 ± 0.08
5 mM GTP	739 ± 186	0.6 ± 0.09
Without nucleotides	170 ± 136	0

^*a*^ ND, not detected. The values represent the mean and variance of three replicate enzyme assays.

### Influence of nucleotides on 2-phenanthroate:CoA ligase activity

Similar to other aryl-CoA ligases, 2-phenanthroate:CoA ligase uses ATP as co-substrate for the conversion of 2-phenanthroic acid to 2-phenanthroyl-CoA and releases AMP (Fig. S3). Replacing ATP by either ADP, CTP, GTP, or UTP revealed 2-phenanthroate:CoA ligase activity with all four nucleotides using 2-phenanthroate:CoA ligase from cell-free extracts (Table 2). A half-maximal activity was observed when ATP was replaced by CTP. Even in the absence of nucleotides, a slight formation of 2-phenanthroyl-CoA was observed probably due to residual ATP present in the cell-free extract.

**Fig 5:**
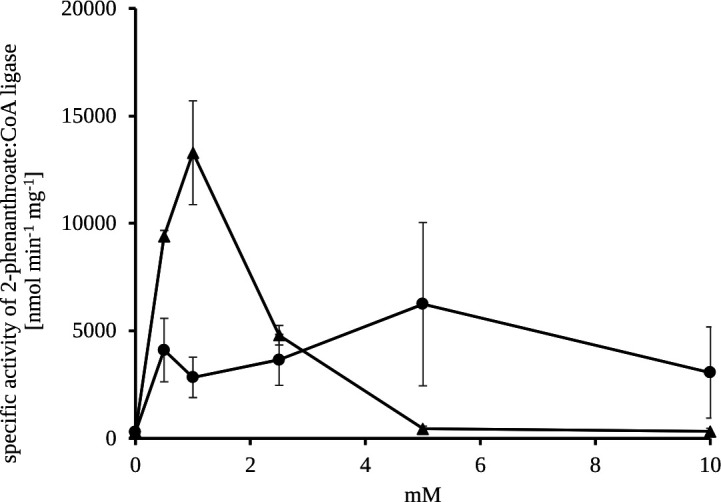
Influence of ATP and ADP on 2-phenanthroate:CoA ligase activity. Production of 2-phenanthroyl-CoA with different concentrations of either ATP (▲) or ADP (●) was applied from 0 to 10 mM and measured after 60 min of incubation at 30°C. The values depict the mean with error bars as mean variance of two replicate enzyme assays.

When ATP was replaced by ADP, production of 2-phenanthroyl-CoA increased 1.2-fold. Comparing different concentrations of ATP and ADP for enzyme activity revealed that 2-phenanthroate:CoA ligase from cell-free extracts is inhibited at ATP concentrations higher than 1 mM (Fig. 5). With increasing ATP concentrations up to 10 mM, the production of 2-phenanthroyl-CoA decreased to 2%, whereas the best conversion of 2-phenanthroic acid with ADP was observed at a concentration of 5 mM (6,245 nmol min^−1^ mg^−1^), which was still half of the activity observed with 1 mM ATP. The purified recombinant 2-phenanthroate:CoA ligase showed 1.3-fold higher specific activity toward ATP than ADP. Moreover, the recombinant enzyme showed 96.6% activity reduction when CTP replaced ATP and showed no activity with UTP or GTP (Table 2). The difference in the performance of the purified recombinant enzyme and the cell-free extract is due to the presence of other aryl-CoA ligases in the cell-free extract, which may behave differently.

### Effect of cations on 2-phenanthroate:CoA ligase activity

**Fig 6:**
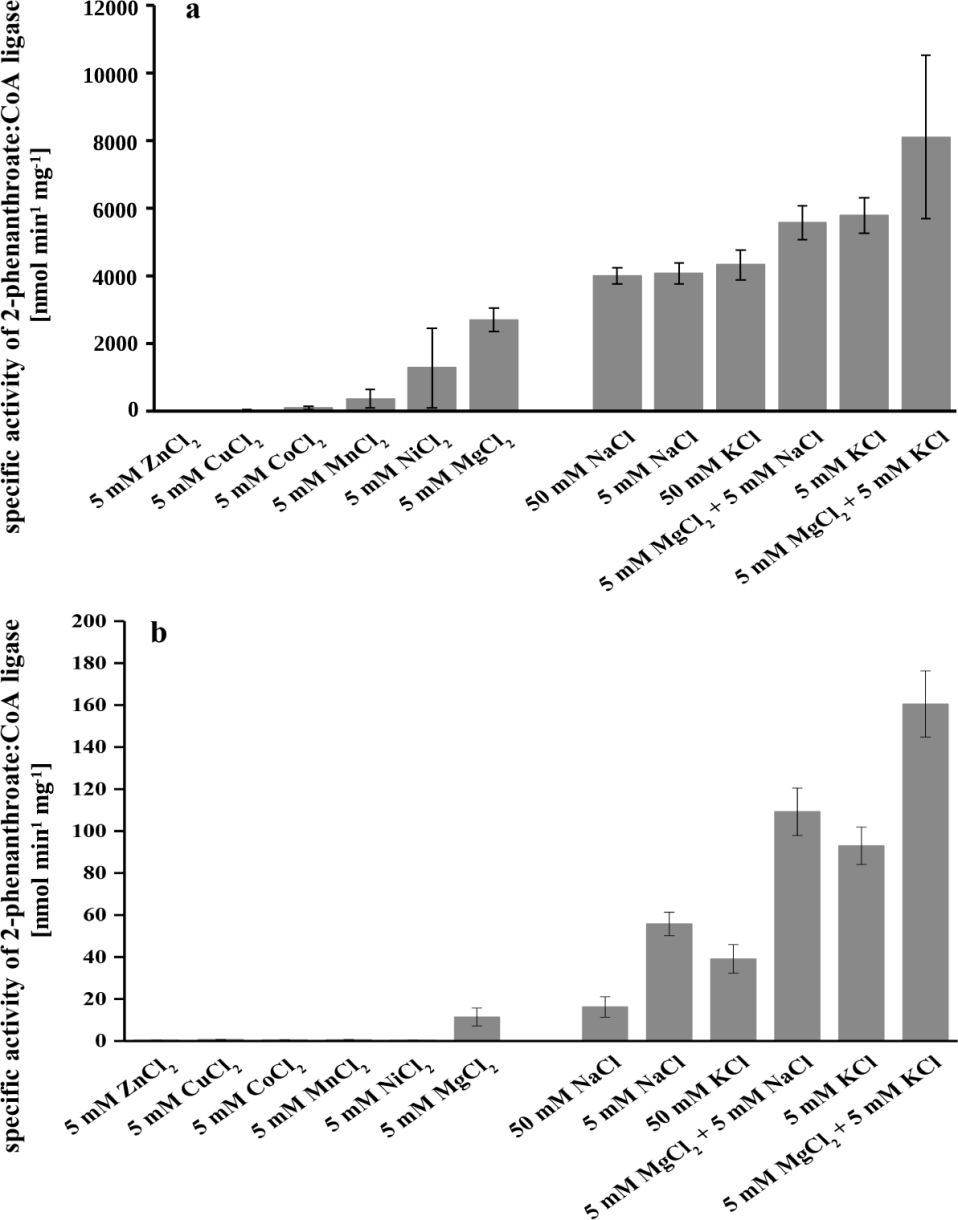
2-Phenanthroate:CoA ligase activity in the presence of divalent cations (left) and monovalent cations or in combination with 5 mM MgCl_2_ (right). Activity was measured using ligase from cell-free extract (a) and the heterologously expressed and purified enzyme (b). 2-Phenanthroyl-CoA production was measured after 60 min of incubation at 30°C. The values depict the mean with error bars as variance of two replicate enzyme assays.

MgCl_2_ was replaced by different cations to determine the effect on 2-phenanthroate:CoA ligase activity from cell-free extracts. Replacing MgCl_2_ with ZnCl_2_, CuCl_2_, and CoCl_2_ resulted in loss of 2-phenanthroyl-CoA production (Fig. 6a). In contrast, performing the ligase assay with the monovalent cations NaCl or KCl resulted in 1.5- to 2-fold higher ligase activity than with MgCl_2_. When MgCl_2_ was combined with 5 mM NaCl the observed ligase activity was twofold higher than with MgCl_2_ only. A combination of 5 mM MgCl_2_ with 5 mM KCl even showed a threefold higher activity. The effect of cations on the purified, recombinant 2-phenanthroate:CoA ligase activity was comparable with the one from cell-free extract (Fig. 6b). MgCl_2_ was the best cation for the highest production of 2-phenanthroyl-CoA. Moreover, 5 mM NaCl or 5 mM KCl showed three- to fivefold higher production of 2-phenanthroyl-CoA than MgCl_2_. The production was even 5.5- and 8-fold higher when MgCl_2_ was combined with 5 mM NaCl and 5 mM KCl, respectively.

### pH optimum and effect of oxygen on 2-phenanthroate:CoA ligase activity

The rates of 2-phenanthroyl-CoA formation at different pH values were determined in anaerobic ligase assays using cell-free extract and aerobic ligase assays using the purified recombinant enzyme with 5 mM MgCl_2_ and 100 mM Tris/HCl as buffer (Fig. S4). A pH optimum was observed at pH 7.5 for phenanthroate:CoA ligase from TRIP culture-cfe, and from *E. coli*. However, 2-phenanthroate:CoA ligase activity was also detected at lower and higher pH values with a half-maximal activity at pH 8.5–9.

Performing the ligase assay under ambient air resulted in the same activity as under anoxic conditions for both the purified enzyme from cell-free extracts or from *E. coli*. This shows that 2-phenanthroate-CoA ligase is oxygen insensitive, similar to other aryl-CoA ligases (Fig. S5).

### Putative CoA-transferase activity of cell-free extracts and purified enzyme with succinyl-CoA or acetyl-CoA

To evaluate a potential CoA-transferase activity in the cell-free extract and the purified recombinant enzyme, free CoA was replaced by acetyl-CoA or succinyl-CoA. Only a minimal production of 2-phenanthroyl-CoA was detected with succinyl-CoA or acetyl-CoA as CoA donor and no reverse reaction could be seen (Table 3). The formation of 2-phenanthroyl-CoA slightly increased with addition of ATP, but was still below the production rate with free CoA. The resulting 2-phenanthroyl-CoA production in the assays with succinyl-CoA or acetyl-CoA is most likely based on the fact that the cell-free extract also contained enzymes that are able to convert succinyl-CoA as well as acetyl-CoA and thus provide free CoA for the aryl-CoA ligase (20). The purified recombinant enzyme supported this evidence, demonstrating the highest production of 2-phenanthroyl-CoA only in the presence of CoA and ATP.

**TABLE 3:** Conversion of 2-phenanthroic acid to 2-phenanthroyl-CoA with different CoA sources^*a*^

Parameter	Specific activity of phenanthroate:CoA ligase (nmol min^−1^ mg^−1^)	
	Cell-free extract	Heterologously expressed
HS-CoA + ATP	7,769 ± 2,211	210 ± 15.9
Succinyl-CoA + ATP	4,274 ± 1,470	70.3 ± 9.1
Succinyl-CoA	79 ± 22	0.46 ± 0.03
Acetyl-CoA + ATP	456 ± 65	4.1 ± 0.09
Acetyl-CoA	102 ± 38	0.63 ± 0.04

^*a*^ The values represent the mean and variance of three replicate enzyme assays.

**Fig 7:**
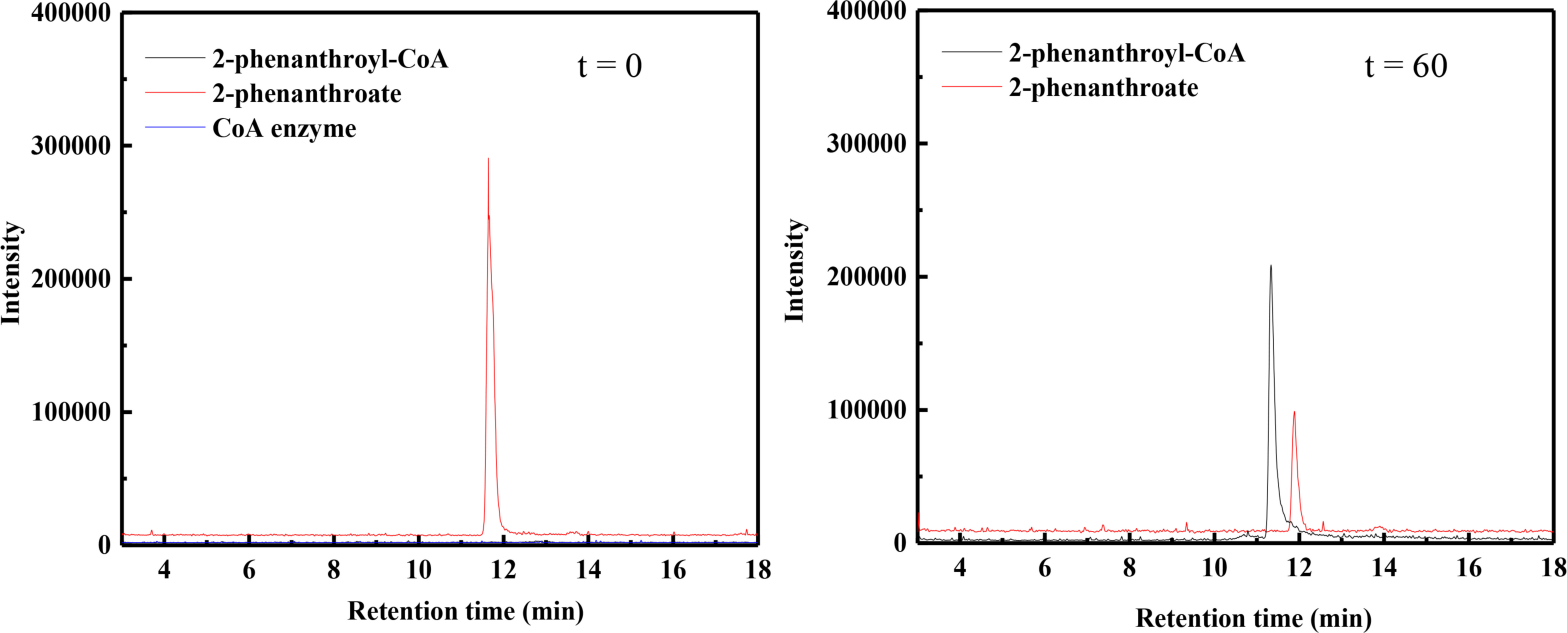
LC-MS analysis showing the time-dependent conversion of 2-phenanthroic acid to 2-phenanthroyl-CoA using the heterologously expressed 2-phenanthroate:CoA ligase. The red line indicates the substrate, 2-phenanthroate (*m/z* = 221, negative ion mode). The gray line indicates the 2-phenanthroyl-CoA produced after 60 min (*m/z* = 972, positive ion mode).

The optimized reaction condition of 2-phenanthroate:CoA ligase activity from cell-free extracts was used to examine the catalytic activity of the purified, heterologously expressed 2-phenanthroate:CoA ligase. 3.71 µM of 2-phenanthroyl-CoA was produced under aerobic condition within 60 min incubation at 30°C (Fig. 7), confirming that the gene PITCH_a1330026 is encoding the 2-phenanthroate:CoA ligase enzyme and 2-phenanthroic acid is the suitable substrate for the ligation reaction. The production of 2-phenanthroyl-CoA (*m/z* 971) was confirmed by observing the mass of the product on the positive ion mode (*m/z* 972) of the MS chromatogram.

## DISCUSSION

Five putative 2-phenanthroate:CoA ligase genes were identified in the candidate *Desulfatiglans* TRIP_1, three of which were also expressed during growth with phenanthrene (20). Purification and identification of 2-phenanthroate:CoA ligase was partially successful, but due to the extremely slow growth of the culture with doubling times of several weeks to months, only a small amount of biomass and protein was available. Proteomic analysis of samples after HIC purification revealed that the 2-phenanthroate:CoA ligase is encoded by the gene PITCH_a1330026, which is located adjacent to the putative phenanthrene carboxylase gene cluster (20). Similar to our enzyme, most of the so far identified aryl-CoA ligases are also encoded by genes located in the vicinity of gene clusters involved in the degradation of the corresponding compounds, with exceptions such as the benzoate:CoA ligase of *Magnetospirillium* sp. (39).

Searching for homologous proteins in the NCBI and PDB protein database identified the ligases phenylacetate:CoA ligases PaaK1 (accession no. B4E7B5) and PaaK2 (accession no. B4EL89) from *B. cenocepacia* J2315, 2-hydroxyisobutyric acid:CoA ligase from *A. tertiaricarbonis* L108 (accession no. I3VE75), and AjiA1 from *Streptomyces* sp. (PDB accession no. 6WUQ) as closest relatives with sequence identities between 23.71% and 31.28%. These proteins belong to the adenylate-forming enzyme family especially to the phenylacetate:CoA ligase-like subgroup. The maximum-likelihood phylogenetic tree of the amino acid sequence of 2-phenanthroate:CoA ligase and several members of the aryl-CoA ligases as well as 2-hydroxyisobutyric acid:CoA ligase and AjiA1 revealed that 2-phenanthroate:CoA ligase belong to the phenylacetate:CoA ligase subgroup, but forms an own branch within this group.

Despite the overall low sequence similarity typical for the class I adenylate-forming enzyme family, the enzymes share a common general architecture with a large N-terminal domain and a small C-terminal domain connected via a short flexible hinge as well as several highly conserved motifs (36). Multiple sequence alignment of the 2-phenanthroate:CoA ligase and several members of the aryl-CoA ligase family allowed us to predict the structure and function of 2-phenanthroate:CoA ligase. Four of the 14 known highly conserved sequence motifs (36), including phosphate-binding loop (motif A3/motif 3), adenine-binding motif (A5), and linker motif (A8), were identified in 2-phenanthroate:CoA ligase sequence.

The linker motif (A8) connects the large N-terminal domain with the smaller C-terminal domain and enables the movement of the C-terminal domain during the CoA-thiosterification reaction (40). This alteration leads to two different interfaces between C-terminal and N-terminal domains that allow to perform two different partial reactions. In the first conformation state, the carboxylic acid can attack the α-phosphate of ATP to form a reactive aryl-AMP intermediate. Afterward, the C-terminal domain rotates by approximately 140° to form the second conformation state, which is required for the formation of the corresponding CoA-thioester (40). The key amino acid involved in the conformation change was determined as D_467_ of this motif (41) which is also conserved in the 2-phenanthroate:CoA ligase sequence. Another quite important motif, the A10 motif, was identified in the 2-phenanthroate:CoA ligase. This conserved motif is located on the C-terminal domain and contains the conserved catalytic residue K_566_. Since the C-terminal domain rotates during the two partial reactions, K_566_ is either present in the active site where it activates the carboxylate group of the substrate and stabilizes the first conformation or is present in solvent in the second conformation (33, 42).

The glycine and serine/threonine-rich phosphate-binding motif was also found in the 2-phenanthroate:CoA ligase sequence. It lies at the interface between the two domains and coordinates the phosphates of ATP during the first conformation as well as stabilizes the C-terminal domain in the second conformation (33, 43). Furthermore, residues that are involved in positioning the ATP such as the R_465_ from motif A8 and D_437_ from motif A7 (motif not found in our alignment) as well as the conserved adenine-binding motif (A5) were identified in the 2-phenanthroate:CoA ligase sequence (40). Since all these mentioned residues and motifs were found in the 2-phenanthroate:CoA ligase sequence we can expect that they fulfill the same function also in 2-phenanthroate:CoA ligase.

Previous investigations conducted on the TRIP culture suggested that the anaerobic degradation mechanism of phenanthrene could be biochemically similar to the anaerobic degradation mechanism of naphthalene (18, 20). The initial step of anaerobic phenanthrene degradation starts with a carboxylation reaction to activate the stable phenanthrene, similar to the degradation process of naphthalene (16, 18, 20). The carboxylation reaction of phenanthrene is catalyzed by a carboxylase enzyme encoded by a gene cluster similar to the ubiD carboxylase genes in both enrichment cultures N47 and NaphS2 (20, 44, 45). The carboxylation reaction of phenanthrene produces phenanthroic acid which is further converted to phenanthroyl-CoA by an ATP-dependent aryl-CoA ligase (18). The production of CoA esters facilitates the further reduction steps of the aromatic rings and enhances product accumulation within the cell, as CoA derivatives cannot penetrate the cell membrane (10, 27). After the aromatic ring reduction, the degradation proceeds further by opening the aromatic rings following β-oxidation-like reactions similar to the same principle of naphthalene degradation (20). In this study, the anaerobic, phenanthrene-degrading, sulfate-reducing enrichment culture TRIP (18, 20) was used to characterize the aryl-CoA ligase reaction for the polycyclic aromatic compound phenanthrene. Due to the slow growth and low biomass of the enrichment culture TRIP all enzyme assays were carried out in cell-free extract rather than with enriched enzyme or with heterologously expressed protein. Even with a dense inoculum of 10%, the culture took about 6–12 months to reach a cell density of approx 5 × 10^6^ cells per mL with a total protein amount of 2.99 ± 1.37 mg. Despite this low protein amount, we were able to measure the activity of phenanthroate:CoA ligases in cell-free extract and to identify and characterize one of these ligases. Moreover, to validate the function and to study the catalytic characteristics of the phenanthroate:CoA ligase, the encoding gene, PITCH_a1330026, was heterologously expressed in *E. coli*, and the enzyme was purified.

By replacing free CoA with succinyl-CoA or acetyl-CoA, we could also exclude a transferase involved in the conversion of phenanthroic acid to phenanthroyl-CoA. The phenanthroate:CoA ligase present in the TRIP culture is highly specific for 2-phenanthroic acid and is only able to convert 3-phenanthroic acid to a minor extent of 20% compared to 2-phenanthroic acid. However, the purified recombinant phenanthroate:CoA ligase only converted negligible amounts of 3-phenanthroic acid. No other isomers of phenanthroic acid were converted by phenanthroate:CoA ligase also indicating that the product of the phenanthrene carboxylation reaction must be 2-phenanthroic acid as the first metabolite in the degradation pathway (18, 20).

All known aryl-CoA ligases couple their CoA-thioesterification reaction to the hydrolysis of ATP forming AMP and PP_i_ (46–50). 2-Phenanthroate:CoA ligase analogously uses ATP as co-substrate for the conversion of 2-phenanthroate to 2-phenanthroyl-CoA. However, ATP concentrations above 1 mM had inhibitory effect on 2-phenanthroate:CoA ligase activity. Substrate inhibition by ATP was so far only known for a benzoate:CoA ligase of *Rhodopseudomonas palustris* that also shows reduced activity at higher concentrations of benzoate (>0.25 mM) and CoA (>1 mM) (46). 2-Phenanthroate:CoA ligase, however, was neither inhibited at increased 2-phenanthroic acid nor CoA concentration (data not shown).

Unusual to most other aryl-CoA ligases, 2-phenanthroate:CoA ligase in cell-free extracts was also active with other nucleotides such as ADP, CTP, UTP, and GTP. Only phenylacetyl:CoA ligase of *Pseudomonas putida* was active when ATP was replaced by ADP, CTP, or UTP (51). However, this could be attributed to the presence of different enzymes in the cell-free extract because the purified recombinant enzyme was only active with the nucleotides ATP and ADP. The purified recombinant enzyme showed 1.3-fold higher specific activity toward ATP than ADP and indicating that the higher activity of the enzyme in cell-free extracts with ADP could be due to the presence of other enzymes. The enzymatic activity of 2-phenanthroate:CoA ligase, from both cell-free extract and recombinant purified enzyme, needed 5 mM MgCl_2_ and 5 mM KCl with the nucleotides ATP and ADP. The higher activity of the recombinant ligase enzyme with ATP compared to ADP is consistent with other ligases, such as the *Aeropyrum pernix* ligase (52). While *A. pernix* ligase demonstrated the ability to utilize ADP as a cofactor, its specific activity was 40% lower when ADP was used as the cofactor, in contrast to ATP. Moreover, acyl-CoA ligase from *Streptomyces aizunensis* demonstrated the ability to utilize ADP but with 50% less product than using ATP (53).

An important factor for proper CoA ligase activity is magnesium since Mg^2+^ forms a complex with ATP and furthermore interacts and stabilizes the acyl-AMP intermediate (29). For most aryl-CoA ligases, Mg^2+^ can be replaced only by Mn^2+^ without loss of activity (46, 47, 51, 54–56). However, 2-phenanthroate:CoA ligase from cell-free extracts showed only 14% of the maximal activity with Mn^2+^ and no activity was observed for the purified recombinant enzyme with Mn^2+^. Other divalent cations such as Zn^2+^, Cu^2+^, Ni^2+^, and Hg^2+^ are known to have strong inhibitory effects on ligase activity which also applied to 2-phenanthroate:CoA ligase, from cell-free extracts and the purified recombinant enzyme, when Mg^2+^ was replaced by Co^2+^, Zn^2+^, or Cu^2+^. Unlike all other aryl-CoA ligases, which were not affected by monovalent cations such as K^+^, Na^+^, Li^+^ and Rb^+^, 2-phenanthroate:CoA ligase showed higher conversion rates with K^+^, Na^+^ or with a combination of 5 mM Mg^2+^ with either 5 mM Na^+^ or 5 mM K^+^. Similar results were only found for xenobiotic/medium-chain fatty acid:CoA ligases of bovine liver mitochondria and cinnamate:CoA ligase of *Hypericum calycinum* cell cultures (57–59). These ligases were active with KCl at an optimum concentration of 50 mM for xenobiotic/medium-chain fatty acid:CoA ligases and 100 mM for cinnamate:CoA ligase. In contrast, 2-phenanthroate:CoA ligase from cell-free extract and the purified recombinant enzyme was less active with 50 mM than with 5 mM KCl. A combination of KCl with MgCl_2_ showed the overall greatest conversion of 2-phenanthroic acid to 2-phenanthroyl-CoA, analogous to the results for xenobiotic/medium-chain fatty acid:CoA ligases and cinnamate:CoA ligase. K^+^ was shown to improve the enzyme affinity toward CoA for xenobiotic/medium-chain fatty acid:CoA ligases, whereas no effect was seen for binding ATP or carboxylic acid (58). 2-Phenanthroate:CoA ligase was also active with Na^+^ but slightly lower than with K^+^. Xenobiotic/medium-chain fatty acid:CoA ligases XL-I and XL-III showed substrate-dependent activity with Na^+^, while cinnamate:CoA ligase was inactive when K^+^ was replaced by Na^+^ (58, 59). K^+^ is maybe preferred by ligases due to its lower charge and larger ionic radius compared to Na^+^. The highest activity of the 2-phenanthroyl-CoA ligase from cell-free extract in all enzymatic assays compared to the recombinant one could be because a part from the recombinant enzyme was expressed as inclusion bodies, which negatively affected the enzyme activity (60).

### Conclusion

In this study, we demonstrate the first characterization of an aryl-CoA ligase involved in the anaerobic degradation of a PAHs. The 2-phenanthroate:CoA ligase of the sulfate-reducing enrichment culture TRIP encoded by the gene PITCH_a1330026 of the candidate *Desulfatiglans* TRIP_1 was purified and characterized.

Properties such as ATP-dependence and oxygen insensitivity of 2-phenanthroate:CoA ligase are similar to other so far characterized aryl-CoA ligases. The requirement of a combination of KCl and MgCl_2_ for good enzyme activity or the inhibitory effect of ATP at higher concentration than 1 mM distinguishes 2-phenanthroate:CoA ligase from other members of this enzyme family. Based on this chemical evidence and the results from the sequence alignment as well as from the constructed maximum-likelihood phylogenetic tree, we conclude that 2-phenanthroate:CoA ligase belongs to the adenylate-forming enzyme family, especially to the phenylacetate:CoA ligase subgroup, but nevertheless forms a new branch within this subgroup. On the basis of multiple sequence alignments, we can predict that this ligase consists of the general two domain structures, with a large N-terminal and a small C-terminal domain. Furthermore, four highly conserved sequence motifs including a phosphate-binding loop, an adenine-binding motif, and the linker motif were identified in 2-phenanthroate:CoA ligase sequence.

## MATERIALS AND METHODS

### Growth of enrichment culture TRIP

The sulfate-reducing enrichment culture TRIP (18, 20) was cultivated under anoxic conditions in 150 mL carbonate-buffered freshwater medium (61) amended with 10 mM sulfate as terminal electron acceptor and 1.2% (wt/vol) phenanthrene dissolved in paraffin oil, as sole carbon and energy source. The headspace was flushed with N_2_/CO_2_ (80:20) and the bottles were closed with blue butyl stoppers (Glasgerätebau Ochs, Göttingen, Germany). Bottles were inoculated with 10% of the previous TRIP culture and incubated at 30°C in the dark until growth became visible as faint turbidity. Common cultivation until the cultures reached a cell density of around 10^6^ cells mL^−1^ was around 6–12 months. About 1 mL of cell extract with a protein content of around 2 mg could be obtained from 1 L of culture.

### Sample preparation for bottom-up proteomics by LC/MS/MS

Samples for LC/MS/MS were digested in-solution. Briefly, 10 µg enriched proteins or whole-cell extracts were precipitated with MeOH/CHCl_3_ (62). The resulting protein pellet was dried on air for 5 min and taken up in 8 M urea containing 100 mM ammonium bicarbonate. Protein reduction and alkylation were performed by adding 5 mM dithiothreitol (30 min, room temperature), followed by 20 mM iodoacetamide (30 min, room temperature). The excess iodoacetamide was quenched by adjusting the dithiothreitol concentration to 30 mM. Samples were then digested with 500 ng LysC for 3 h at 37°C. After adjusting the urea concentration to 0.8 M, the samples were digested for 16 h with 500 ng trypsin at 37°C. The digestion was stopped by adding formic acid to a final concentration of 0.5%. The supernatant containing the digestion products was passed through homemade glass microfiber stage tips (GE Healthcare, pore size 1.2 µM, thickness 0.26 mm). Cleared tryptic digests were then desalted on homemade C18 stage tips as described before (63). Briefly, peptides were immobilized and washed on a two-disc stage tip. After elution from the stag tips, samples were dried using a vacuum concentrator (Eppendorf) and the peptides were taken up in 0.1% formic acid solution (10 µL).

### LC-MS/MS settings for peptide analysis

Experiments were performed with an Orbitrap Fusion LUMOS instrument (Thermo) that was coupled to an EASY-nLC 1200 liquid chromatography (LC) system (Thermo). The LC was operated in the one-column mode. The analytical column was a fused silica capillary (75 µm × 44 cm) with an integrated emitter (ESi Source Solutions) packed in-house with Reprosil-Pur 120 C18-AQ 1.9 µm resin (Dr. Maisch). The analytical column was encased by a column oven (Sonation PRSO-V2) and attached to a nanospray flex ion source (Thermo). The column oven temperature was set to 50°C during data acquisition. The LC was equipped with two mobile phases: solvent A (0.1% formic acid, FA, in water) and solvent B (0.1% FA in 80% acetonitrile, ACN). All solvents were of UPLC grade (all UHPLC solvents: Honeywell Specialty Chemicals, Seelze, Germany). Peptides were directly loaded onto the analytical column with a maximum flow rate that would not exceed the set pressure limit of 980 bar (usually around 0.5–0.6 µL/min). Peptides were subsequently separated on the analytical column by running a 200 min gradient of solvent A and solvent B (start with 10% B; gradient 10–40% B for 170 min; gradient 40–100% B for 20 min and 100% B for 10 min) at a flow rate of 250 nL/min. The mass spectrometer was controlled by the Orbitrap Fusion Lumos Tune Application (version 3.3.2782.28) and operated using the Xcalibur software (version 4.3.73.11). The mass spectrometer was set in the positive ion mode. Precursor ion scanning was performed in the Orbitrap analyzer (FTMS; Fourier Transform Mass Spectrometry) in the scan range of *m/z* 375–1,700 and at a resolution of 240,000 with the internal lock mass option turned on (lock mass was 445.120025 *m/z*, polysiloxane) (64). Product ion spectra were recorded in a data-dependent fashion in the Orbitrap (FTMS) in a variable scan range and at a resolution of 7,500. The ionization potential (spray voltage) was set to 2.3 kV. Peptides were analyzed using a “Top Speed” regime [repeating cycle of full precursor ion scan (AGC target “standard” or 50 ms) followed by dependent MS2 scans for 3 s (minimum intensity threshold 2 × 10^4^)]. The precursor ions were isolated using the quadrupole (isolation window 1.2 *m/z*). After Higher-energy C-trap dissociation (HCD) (normalized collision energy set to “fixed” and 33%), the product ions were measured in the FTMS (AGC target “standard,” acquisition time “auto” and mass range “auto”). During MS2 data acquisition, dynamic ion exclusion was set to 30 s. Only charge states between 2 and 7 were considered for fragmentation.

### Peptide and protein identification using MaxQuant

RAW spectra were submitted to an Andromeda (65) search in MaxQuant (1.6.10.43) using the default settings (66). Label-free quantification and match-between-runs were activated (67). The search was performed against the following in-house generated databases: TRIP_1 candidate Desulfatiglans.fasta (4,866 entries), TRIP_2 candidate Desulfatiglans.fasta (4,891 entries), TRIP_3 candidate Paludibacter.fasta (2,636 entries), TRIP_4 candidate Spirochaete.fasta (2,930 entries), and TRIP_5 candidate Zixibacteria.fasta (3,045 entries) (20). All searches included a contaminants database search (as implemented in MaxQuant, 245 entries). The contaminants database contains known MS contaminants and was included to estimate the level of contamination. Andromeda searches allowed oxidation of methionine residues (16 Da) and acetylation of the protein N-terminus (42 Da) as dynamic modifications and the static modification of cysteine (57 Da, alkylation with iodoacetamide). Enzyme specificity was set to “Trypsin/P” with two missed cleavages allowed. The instrument type in Andromeda searches was set to Orbitrap and the precursor mass tolerance was set to ±20 ppm (first search) and ±4.5 ppm (main search). The MS/MS match tolerance was set to ±0.5 Da. The peptide spectrum matches FDR and the protein FDR was set to 0.01 (based on target-decoy approach). Minimum peptide length was seven amino acids. For protein quantification unique and razor peptides were allowed. Modified peptides were allowed for quantification. The minimum score for modified peptides was 40. Label-free protein quantification was switched on, and unique and razor peptides were considered for quantification with a minimum ratio count of 2. Retention times were recalibrated based on the built-in nonlinear time-rescaling algorithm. MS/MS identifications were transferred between LC-MS/MS runs with the “match between runs” option in which the maximal match time window was set to 0.7 min and the alignment time window set to 20 min. The quantification is based on the “value at maximum” of the extracted ion current. At least two quantitation events were required for a quantifiable protein. Further analysis and filtering of the results were done in Perseus v1.6.10.0 (68).

### Sequence comparison of 2-phenanthroate:CoA ligase

Homologous proteins of 2-phenanthroate:CoA ligase were searched in the NCBI and PDB databases via the program BLAST. Amino acid sequences of 2-phenanthroate:CoA ligase, its homologous proteins and 14 representative aryl-CoA ligases were aligned using ClustalW algorithm in the program Mega-X (37). The phylogenetic tree was constructed using the maximum-likelihood method and JTT matrix-based model (38) in Mega-X.

### Purification of phenanthroate:CoA ligase

Phenanthroate:CoA ligase was enriched from a 1.5 L TRIP culture. Cells were resuspended in 1.6 mL 100 mM Tris/HCl, pH 7.3 and broken with a French press (Thermo Electron, Waltham, USA) at a pressure of 6.9 MPa. Cell debris and unbroken cells were removed by centrifugation at 16,000 × *g*, 4°C for 30 min.

The purification procedure included ammonium sulfate precipitation and HIC. Due to the low protein amounts, no further purification steps were possible.

About 1.0 mL of the soluble protein fraction was treated with 55% ammonium sulfate (2.5 M end concentration), stirred for 30 min on ice, and centrifuged at 16,060 × *g*, 4°C for 30 min. The pellet containing phenanthroate:CoA ligase activity was resuspended in 50 mM sodium phosphate buffer, pH 7.0 containing 2 M ammonium sulfate and applied to high-performance liquid chromatography (HPLC) (ÄKTAmicro, GE healthcare Freiburg, Germany) equipped with a reversed-phase column (HiTrap Butyl HP, 1 mL, GE healthcare, Freiburg, Germany). The column was equilibrated with 50 mM Na_3_PO_4_ buffer, pH 7.0 followed by 50 mM Na_3_PO_4_, pH 7.0 containing 2 M ammonium sulfate. The protein solution was loaded on the column and after washing the column with the same buffer, the ligase was eluted with a linear gradient of 2 M to 0 M ammonium sulfate at a flow of 0.3 mL min^−1^. Fractions containing phenanthroate:CoA ligase activity were concentrated with an IVSS VIVASPIN concentrator (cutoff 10,000 MW, GE Healthcare, Freiburg, Germany) for 30 min at 16,060 × *g*, 4°C and washed two times with 100 mM Tris/HCl, pH 7.3 under the same conditions. The concentrate was resuspended in 200 µL 100 mM Tris/HCl, pH 7.3. Furthermore, sodium dodecyl sulfate-polyacrylamide gel electrophoresis (SDS-PAGE) was performed using Mini-PROTEAN TGX precast gels (Bio-Rad Laboratories GmbH, München, Germany) according to Laemmli (69). PageRuler Broad Range unstained Protein Ladder (Thermo Scientific, Waltham, USA) was used as a molecular mass marker. Protein bands were visualized by silver staining (Thermo Fisher Scientific, Waltham, USA).

### Heterologous expression of the gene encoding 2-phenanthroate:CoA ligase

The gene PITCH_a1330026 was cloned into the PASG-IBA103 expression plasmid according to the manual provided by IBA Lifesciences GmbH, Germany. KAPA HiFi DNA Polymerase (Roche) was used to amplify the gene PITCH_a1330026 using the extracted genomic DNA of culture TRIP1 as DNA template with the forward primer AGCGCGTCTCCAATGACTCTCTTTGCCTTAAATTCAG and reverse primer AGCGCGTCTCCTCCCTGGATGTGATTTTGAAATGGATG. The restriction enzyme Esp3I was used to clone the fragment into the vector (restriction site is underlined). The PCR program used for gene amplification was as follows: 95°C (3 min), 35 cycles of 95°C (30 s), 54.5°C (60 s), and 72°C (60 s), then a final extension for 5 min at 72°C. Afterward, 2 nM of the PCR product was ligated with 5 ng of PASG-IBA expression plasmid and transformed to NEB 5-alpha competent *E. coli* according to the provided manual by IBA Lifesciences GmbH. The recombinant plasmid was extracted from NEB 5-alpha competent *E. coli* and then transformed into BL21(DE3) competent *E. coli*, and the successful gene cloning was confirmed by plasmid sequencing. The cells were grown with LB medium containing 100 µg/mL ampicillin at 37°C under shaking and then induced by adding 0.2 µg/mL anhydrotetracycline and the incubation temperature reduced to 20°C when OD_550_ reached 0.6. The cells were harvested after overnight incubation and resuspended in 1 mL of buffer W (100 mM Tris/HCl, pH 7.8, and 150 mM NaCl) per 1 g wet cells. After a single passage through a cooled French pressure cell to disrupt the cells at 1,100 psi, cell debris was removed by centrifugation at 16,000 × *g*, 4°C for 45 min. The produced supernatant was subjected to streptactin affinity resin (IBA Lifesciences, Germany) equilibrated with buffer W and the unbound proteins were washed eight times using buffer W and the phenanthroate:CoA ligase was eluted by buffer BXT (buffer W + 50 mM biotin).

### Determination of phenanthroate:CoA ligase activity and responsible cofactors

All enzyme assays were performed under strictly anoxic conditions in duplicates, except when testing the influence of oxygen on ligase activity. Separation of the aqueous culture supernatant from the paraffin oil was performed inside a glove box (M. Braun Intergas-Systeme GmbH, Garching, Germany). The cells were harvested in air-tight beakers by centrifugation at 3,100 × *g*, 4°C for 30 min. The cell pellet was washed either with 100 mM MOPS/KOH, pH 7.3, 15 mM MgCl_2_ or 100 mM Tris/HCl, pH 7.3 before resuspending in 1.6 mL per 1.5 L culture volume, of the same buffer. The cells were opened with a French Press (Thermo Electron, Waltham, USA) operated at 6.9 MPa. To remove all cell debris, crude cell extract was centrifuged at 16,000 × *g*, 4°C for 30 min and the protein-containing supernatant was used for the ligase assays.

Ligase activity was examined from the partially purified ligase enzyme from cell-free extracts of culture TRIP1, and from the purified recombinant phenanthroate:CoA ligase. Phenanthroate:CoA ligase activity was measured with the partially purified ligase enzyme from cell-free extract of culture TRIP1 or the purified, recombinant enzyme with a buffer containing 5 mM ATP, 1 mM CoA-SH, 2 mM DTT, 1 mM 2-phenanthroic acid, 5 mM MgCl_2,_ 5 mM KCl, and 100 mM Tris/HCl buffer, pH 7.3. The reaction was started by the addition of 40% (vol/vol) cell-free extract (200 µL reaction volume) with a protein concentration between 0.12 and 0.22 mg, and for the purified heterologously expressed phenanthroate:CoA ligase by addition of 0.2 mg protein. Before starting the reaction by adding the purified recombinant phenanthroate:CoA ligase, the purified enzyme must be concentrated to 100 µL volume with 0.2 mg protein concentration. Subsequently, the enzymatic assay with the purified recombinant phenanthroate:CoA ligase contains 100 µL buffer with the previously mentioned cofactors plus 100 µL pure concentrated enzyme. Purified protein concentration was performed using 10 kDa molecular weight cutoff membrane. The enzyme assays were incubated for 60 min at 30°C and 900 rpm in a Thermomix Block (ThermoMixer C, Eppendorf, Germany). Directly after 60 min incubation, the enzymatic reaction was stopped by adding a double volume of methanol, samples were centrifuged at 16,000 × *g*, 4°C for 1 h, and the supernatant was used for detecting the concentration of the produced 2-phenanthroyl-CoA using LC-MS (18).

Substrate specificity of phenanthroate:CoA ligase from TRIP1 culture-cfe and the recombinant enzyme was investigated using 100 mM MOPS/KOH buffer, pH 7.3, 5 mM MgCl_2_ and the substrates 1-phenanthroic acid (Toronto Research Chemicals), 2-phenanthroic acid (Sigma Aldrich), 3-phenanthroic acid (Toronto Research Chemicals), 4-phenanthroic acid (Sigma Aldrich), or 9-phenanthroic acid (Toronto Research Chemicals).

Succinyl-CoA or acetyl-CoA dependent conversion of 2-phenanthroic acid to 2-phenanthroyl-CoA was examined with 100 mM Tris/HCl pH 7.3, 5 mM acetyl-CoA or succinyl-CoA, 1 mM 2-phenanthroic acid, 2 mM DTT, 5 mM MgCl_2,_ 5 mM KCl, and with or without 5 mM ATP. In addition, the reverse reaction was tested using 1 mM acetate or succinate, 1 mM 2-phenanthroyl-CoA, 2 mM DTT, and 5 mM MgCl_2_.

The effect of pH on the production of 2-phenanthroyl-CoA was analyzed with a glycine-HCl buffer (100 mM) in the pH range 3–5, Tris/HCl buffer (100 mM) in the pH range 6–8, and glycine-NaOH buffer (100 mM) in the pH range 9–11.

### LC-MS settings for small molecule analysis

Samples were analyzed with an LC-2040C system coupled to an LC-MS-2020 single quadrupole mass-spectrometer (Shimadzu Deutschland, Duisburg, Germany) and eluted in a gradient. Analytes were separated on a Nucleodur C18 Gravity-SB column, 100 × 3 mm^2^, 5 µm particle size (Macherey-Nagel, Dueren, Germany) at 35°C. MS analysis was performed with an ESI system in single ion negative and positive mode. The voltage of the ESI system was either −4.5 kV, negative ion mode, or 4.5 kV, positive ion mode at a temperature of 350°C. Nebulizing gas flow was set to 1.5 L min^−1^ and drying gas flow was 12 L min^−1^. The heat block had a temperature of 200°C and the desolvation line was set to 0 V and 250°C. 2-phenanthroyl-CoA was detected in positive ion mode with an RF voltage of 120 V and AMP was measured in negative ion mode with an RF voltage of 52 V.

For measuring 2-phenanthroyl-CoA, 0.1% (wt/vol) ammonium formate was used as eluent A and acetonitrile as eluent B. The acetonitrile concentration was ramped from 10% to 50% over 18 min at a flow rate of 0.7 mL min^−1^. AMP was measured with water, methanol, and acetonitrile as eluents starting from 88% water, 4.5% methanol, and 7.5% acetonitrile in a linear gradient ramping to 60% water, 2.5% methanol, and 37.5% acetonitrile within 3 min.

## Data Availability

The mass spectrometry bottom-up proteomics data for the on-bead digestions are deposited to the ProteomeXchange Consortium via the PRIDE (70) partner repository (https://www.ebi.ac.uk/pride/archive/) with the data set identifier PXD029438.

## SUPPLEMENTAL MATERIAL

Supplemental material aem.01296-24-s0001.docx Figures S1 to S5.

ASM does not own the copyrights to Supplemental Material that may be linked to, or accessed through, an article. The authors have granted ASM a non-exclusive, world-wide license to publish the Supplemental Material files. Please contact the corresponding author directly for reuse.
