# Supplementary material for: Characterization of 2-phenanthroate:CoA ligase from the sulfate-reducing, phenanthrene-degrading enrichment culture TRIP
Source: Appl Environ Microbiol. 2024 Sep 9;90(10):e01296-24. doi: 10.1128/aem.01296-24 (PMC11497795; doi:10.1128/aem.01296-24)
Supplement: Supplemental material — Figures S1 to S5. [file aem.01296-24-s0001.docx]

**Supplementary material**


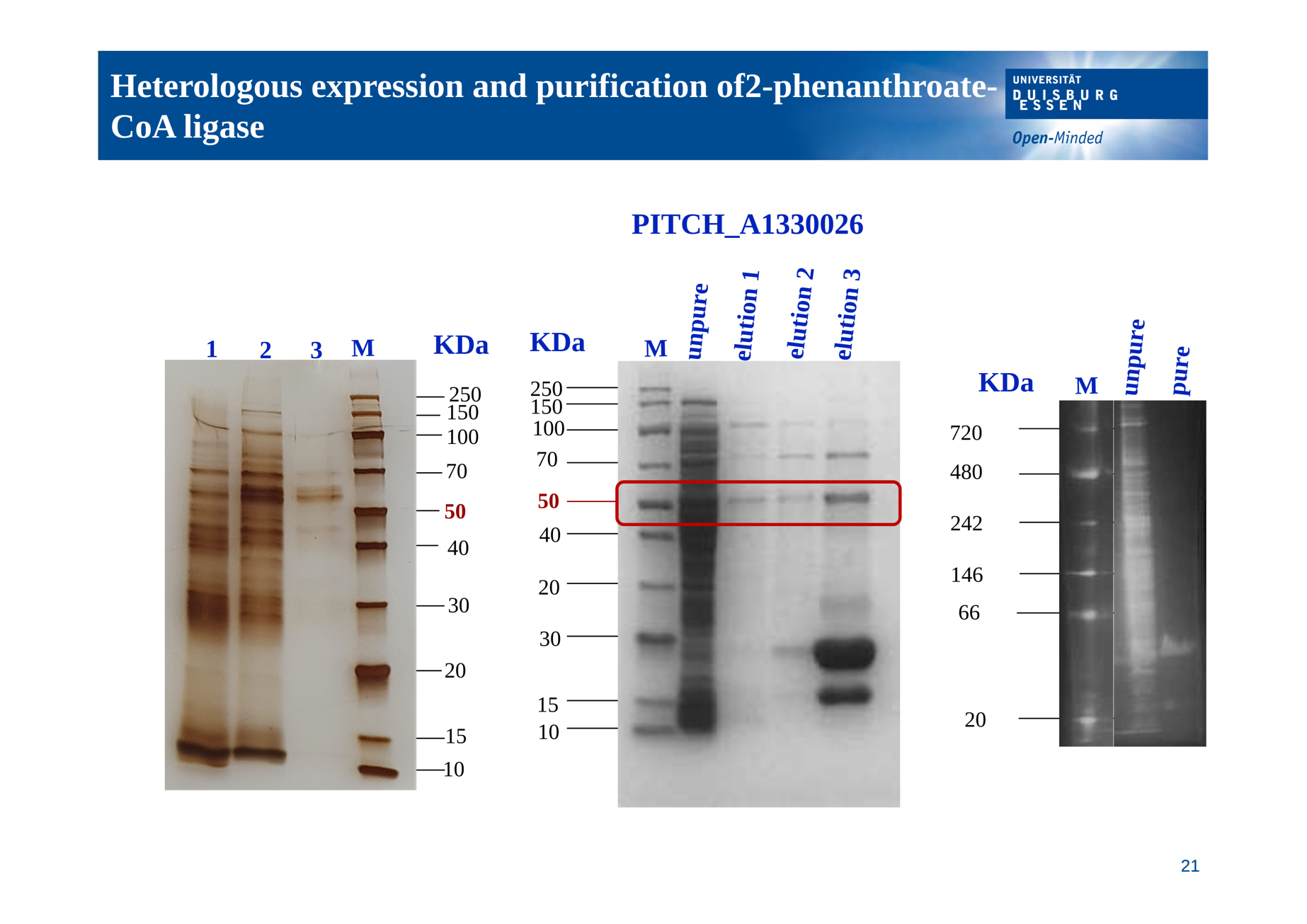


**Fig. S1.** Silver stained SDS-PAGE gel of the purification steps. Lane 1, cell free extract; lane 2, after precipitation with 2.5 M ammonium sulfate; lane 3, after purification with HIC.

**Fig. S2** Multiple alignment of 2-phenanthroate:CoA ligase, 16 representative aryl-CoA ligases, 2‑hydroxyisobutyryl-CoA synthetase, and AjiA1. Conserved structural motifs are highlighted in gray, amino acids that are 100 % conserved within the alignment are depicted violet, amino acids conserved to 70 % are written in blue and amino acids that are 100 % conserved exclusively within the phenylacetate-CoA ligase-like enzyme family are shown in orange.


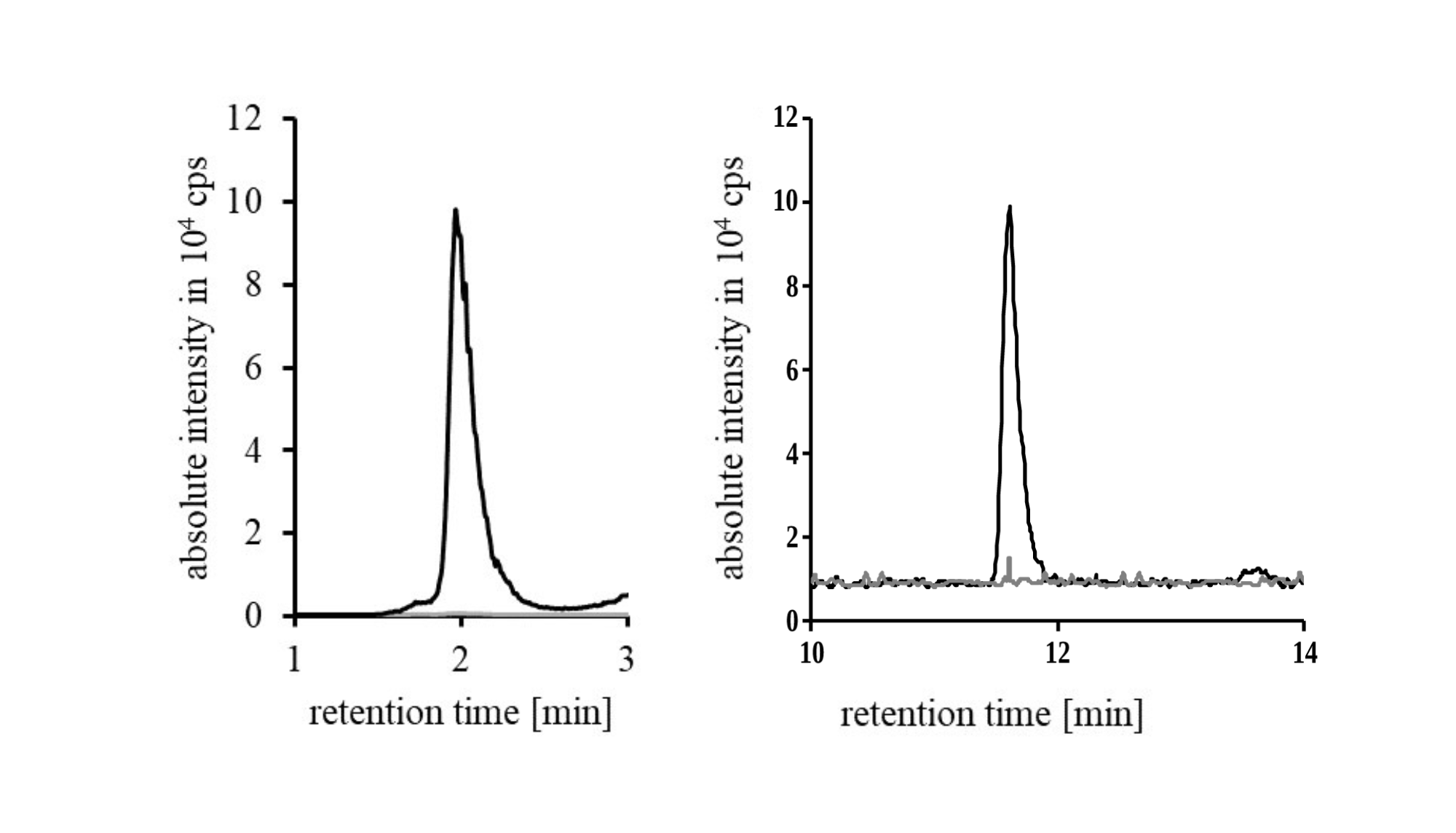


**Fig. S3** LC-MS measurements showing the production of AMP (A) and 2-phenanthroyl-CoA (B) over time. Production of AMP and 2-phenanthroyl-CoA was determined in an anaerobic ligase assay with 5 mM ATP, 1 mM 2-phenanthroic acid, 1 mM SH-CoA, 2 mM DTT, Tris/HCl buffer pH 7.3, 5 mM MgCl_2_ and 40% cell free extract. [grey line: t = 0 min; black line: t = 60 min]


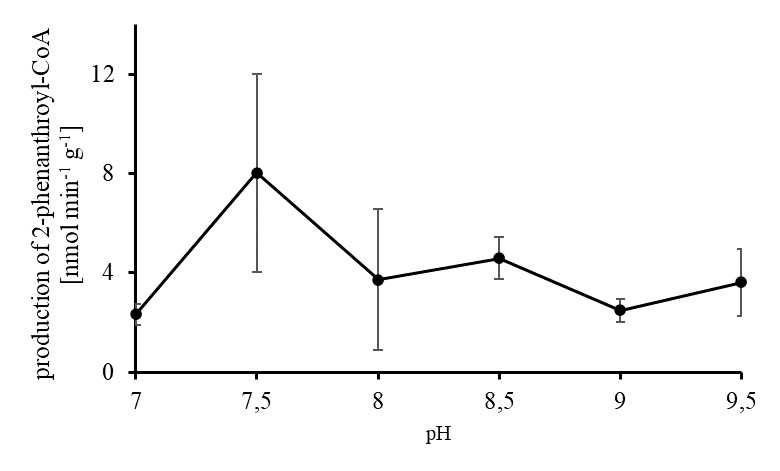


**b**

**a**

**Fig. S4** 2-Phenanthroate:CoA ligase activity at different pH values using TRIP culture-cfe (a) and the purified recombinant enzyme (b). 2-Phenanthroyl-CoA formation was determined in an anaerobic ligase assay with 5 mM ATP, 1 mM 2-phenanthroic acid, 1 mM SH-CoA, 2 mM DTT, 5 mM MgCl_2_ and100 mM Tris/HCl after 60 min of incubation at 30 °C. The values depict the mean with error bars as mean variance of two replicate enzyme assays.


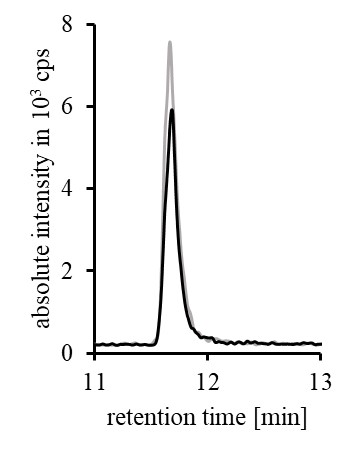


**Fig. S5** LC-MS measurements showing the production of 2-phenanthroyl- CoA during anaerobic and aerobic ligase assay. 2-Phenanthroate:CoA ligase activity was measured under unoxic [black] and oxic [grey] conditions using 5 mM ATP, 1 mM 2-phenanthroic acid, 1 mM SH-CoA, 2 mM DTT, Tris/HCl buffer pH 7.3, 5 mM MgCl_2_ and 40% cell free extract after 60 min
